# Formulation and Characterization of O/W Nanoemulsions of Hemp Seed Oil for Protection from Steatohepatitis: Analysis of Hepatic Free Fatty Acids and Oxidation Markers

**DOI:** 10.3390/ph15070864

**Published:** 2022-07-14

**Authors:** Mona Qushawy, Yasmin Mortagi, Reem Alshaman, Hatem I. Mokhtar, Fatma Azzahraa Hisham, Abdullah Alattar, Dong Liang, Eman T. Enan, Amira H. Eltrawy, Zainab H. Alamrani, Sara A. Alshmrani, Sawsan A. Zaitone

**Affiliations:** 1 Department of Pharmaceutics, Faculty of Pharmacy, University of Tabuk, Tabuk 71491, Saudi Arabia; mqushawy@ut.edu.sa or; 2 Department of Pharmaceutics, Faculty of Pharmacy, Sinai University, Alarish 45511, North Sinai, Egypt; yasmin.mohamed@su.edu.eg; 3 Department of Pharmacology and Toxicology, Faculty of Pharmacy, University of Tabuk, Tabuk 71491, Saudi Arabia; ralshaman@ut.edu.sa (R.A.); aalattar@ut.edu.sa (A.A.); 4 Department of Pharmaceutical Chemistry, Faculty of Pharmacy, Sinai University, Kantara Branch, Ismailia 41636, Egypt; 5 Department of Medical Biochemistry and Molecular Biology, Faculty of Medicine, Mansoura University, Mansoura 35516, Egypt; fatmahisham@mans.edu.eg; 6 Department of Pharmaceutical & Environmental Health Sciences, College of Pharmacy and Health Sciences, Texas Southern University, Houston, TX 77004, USA; dong.liang@tsu.edu; 7 Department of Pathology, Faculty of Medicine, Mansoura University, Mansoura 35516, Egypt; emanenan@mans.edu.eg; 8 Department of Anatomy, Faculty of Medicine, University of Tabuk, Tabuk 71491, Saudi Arabia; aaltrawi@ut.edu.sa; 9 Department of Anatomy and Embryology, Faculty of Medicine, Alexandria University, Alexandria 21526, Egypt; 10 PharmD Program, Faculty of Pharmacy, University of Tabuk, Tabuk 71491, Saudi Arabia; zainabalamrani@gmail.com (Z.H.A.); saralaiwai@outlook.com (S.A.A.); 11 Department of Pharmacology and Toxicology, Faculty of Pharmacy, Suez Canal University, Ismailia 41522, Egypt

**Keywords:** characterization, hemp seed oil, free fatty acids, O/W nanoemulsion, rat NASH, oxidation markers

## Abstract

Non-alcoholic steatohepatitis (NASH) is a common type of metabolic liver disease which is characterized by fatty changes associated with hepatocyte injury, lobular inflammation, and/or liver fibrosis. Nanoemulsions are kinetically stable colloidal systems characterized by small droplet size. Hemp seed oil is a natural oil derived from Cannabis sativa seeds. The current study was designed to formulate nanoemulsion preparations of hemp seed oil with promising enhanced biological activity against high fat (HF) diet induced NASH in rats. Four nanoemulsion formulas (NEFs) were formulated based on high-pressure homogenization technique and evaluated for droplet size, zeta potential (ZP), polydispersity index (PDI), electrical conductivity, pH, and viscosity, as well as the preparation stability. The best NEF was selected to perform an in vivo rat study; selection was based on the smallest droplet size and highest physical stability. Results showed that NEF#4 showed the best physiochemical characters among the other preparations. Twenty male rats were assigned to four groups as follows: normal, NASH control, NASH + hemp seed oil and NASH + hemp seed oil NEF4. The rats were tested for body weight (BWt) change, insulin resistance (IR) and hepatic pathology. The hemp seed NEF#4 protected against NASH progression in rats and decreased the % of BWt gain compared to the original Hemp seed oil. NEF#4 of Hemp seed oil showed greater protective activity against experimental NASH and IR in rats. Hence, we can consider the nanoemulsion preparations as a useful tool for enhancing the biological action of the hemp seed oil, and further studies are warranted for application of this technique for preparing natural oils aiming at enhancing their activities.

## 1. Introduction

Nanoemulsions are preparations which have essential applications for delivering nutraceuticals in the food industry [1,2,3]. The structure and composition of nanoemulsions can be influenced for efficient delivery of biologically active lipophilic compounds [4]. Hemp seed (*Cannabis sativa*) oil is found in the seeds of the hemp plant. Its color is usually dark to clear green with a nutty flavor. Hemp seed oil is usually utilized as a dietary supplement and a food oil [5]. It consists of polyunsaturated fatty acids (PUFAs, 76%), containing ω-3-fatty acids: 54% linolenic acid (https://en.wikipedia.org/wiki/Linoleic_acidLA, accessed on 10 May 2022), 17% α-linolenic acid (ALA), 3% γ-linolenic acid (GLA), 2% and stearidonic acid; both LA and ALA are essential fatty acids. Furthermore, hemp seed oil consists of 5–11% monounsaturated fats and 5–7% saturated fats [6]. From a biological point of view, hemp seed oil has several benefits for health, including inhibition of tumor growth, decreasing the level of cholesterol, and anti-inflammatory effects. In addition, it helps in treating Alzheimer’s disease and cardiovascular diseases. It is characterized by high solubility with water, more so than other vegetable oils, which makes it susceptible to being easily emulsified by emulsifiers [7]. 

Nanoemulsions are dosage forms utilized for delivering active ingredients to target sites. They contain an aqueous phase and an oil phase in addition to an emulsifier. Nanoemulsion droplet sizes range from 10 to 1000 nm [8]. The Brownian motion of small droplets is sufficient for overcoming the low gravitational characters leading to adequate physical stability. We have water-in-oil (W/O) and oil-in-water (O/W) nanoemulsions; the second type is more popular than the first one in pharmaceutical manufacturing as a consequence of its compatibility with water and safety [9].

The liver regulates serum lipids via modulation of lipid biosynthesis and metabolism in addition to lipoprotein reuptake and export mechanisms. Dysregulation in lipid metabolism triggers non-alcoholic fatty liver disease (NAFLD) [10]. Importantly, NAFLD occurs in a multi-phasic process characterized by extensive accumulation of fats with no history of alcohol consumption [11]. NAFLD starts as simple steatosis and may progress to nonalcoholic steatohepatitis (NASH) [12]. NASH describes an advanced lipid accumulation in at least 5% of the hepatocytes, and is characterized by hepatitis and fibrosis. NASH may further develop into life-threatening cirrhosis and hepatic carcinoma; this scenario creates an emerging reason for liver transplants [13]. In NASH, mitochondrial dysfunction produces excessive amounts of reactive oxygen species (ROS) and cytokines, which results in hepatitis. High levels of hepatic free fatty acids (FFAs) are toxic and agents of destruction to the liver [14]. FFA disposal takes place through various mechanisms, but the major route is the triglyceride secretion into the space of DISSE as VLDL [15].

Sirtuins are important modulators of energy metabolism and stress resistance [16]. Sirtuin 1 (SIRT-1) is a master mediator in metabolism and is involved in controlling processes of lipid metabolism such as adipocyte generation and fatty acid synthesis and oxidation. Activation of SIRT-1 is beneficial for protection from NAFLD through inhibiting lipogenesis [17] and mitigation of oxidative stress [18]. SIRT-1 activates a cascade of events, leading to suppression of fat synthesis and fatty acid oxidation stimulation [19] (https://www.frontiersin.org/articles/10.3389/fphar.2018.00201/full, accessed on 10 May 2022). SIRT-1 expression is inhibited in high-fat (HF)-diet-induced obese rodents leading to metabolic liver damages [20]. Lack of SIRT-1 is deleterious to cells, decreases viability, and results in defective metabolism [21]. 

There is accumulating evidence that PUFAs and phytosterols protect against NAFLD [22]. Since hemp seed oil contains PUFAs, high concentrations of phytosterols and essential fatty acids [23], it looks to be a promising phytotherapy against NASH. Thus, in this study, the ameliorative action of a nanoemulsion preparation of hemp seed oil against experimental NASH was investigated. We examined its effectiveness in improving the liver pathology, lipid dysregulation and oxidative stress.

## 2. Results and Discussion

### 2.1. Hemp Seed Oil Characterization

**Figure 1:**
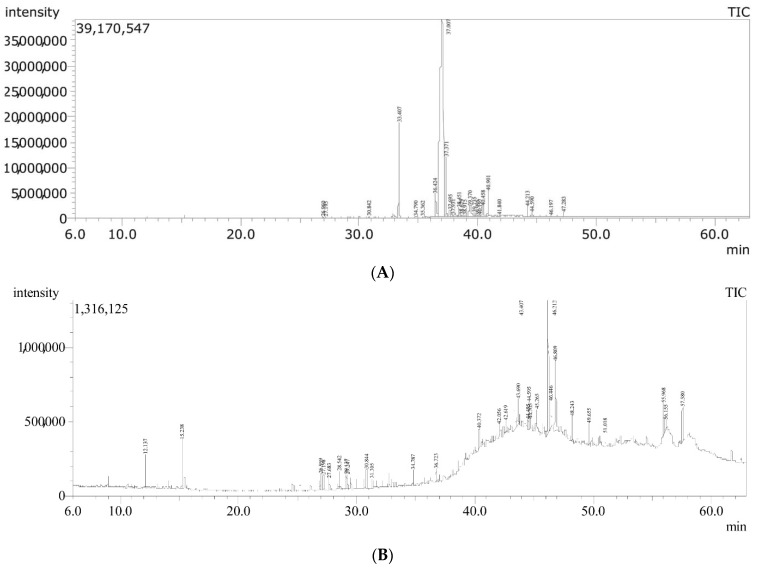
Total ion chromatograms (TICs) of GC-MS/MS analysis of hemp seed oil working sample: (**A**) TIC of saponifiable matter, (**B**) TIC of unsaponifiable matter.

Recorded GC-MS chromatograms and spectra of both unsaponifiable matter and saponifiable fatty acid methyl esters in saponifiable matter (Figure 1A,B) revealed the compositional features of the working sample of hemp seed oil described herein.

Regarding the saponifiable part of the oil, esters of ω-3 PUFAs were determined as follows (ester, % *w*/*w* from saponifiable matter): 9,12,15-octadecatrienoic acid methyl ester (Z,Z,Z) (α-methyl linolenate), 73.7%; γ-methyl linolenate, 3.98%; *cis*-11-eicosenoic acid methyl ester, 0.98%; as well as minor quantities of 7,10,13-eicosatrienoic acid methyl ester and (9Z,11E,13E)-octadeca-9,11,13-trienoic acid methyl ester. Other saturated and unsaturated fatty acid methyl esters were determined at lower proportions (Table 1). γ-Sitosterol was determined to constitute about 4.95% of the unsaponifiable part. Other compounds were also extracted as described (Table 2). 

### 2.2. Preparation of Hemp Seed Nanoemulsion

Hemp seed oil nanoemulsion formulations (NEFs) were prepared using the high-pressure homogenization technique. Four NEFs were prepared according to the composition represented in Table 3. The NEFs were examined with respect to droplet size, polydispersity index (PDI), zeta potential (ZP), electrical conductivity, pH, and viscosity. 

### 2.3. Effect of Surfactant Concentration on the Droplet Size, PDI, and ZP

The droplet size of the prepared hemp seed NEFs ranged from 80.7 ± 3.96 nm for NEF#4 to 140.9 ± 5.62 nm for NEF#1 (Table 4). It was found that the droplet size was affected by the surfactant concentration. The droplet size decreased with increasing surfactant concentration. These results may be due to the decrease in the interfacial tension between the aqueous and oily phases related to the increase in the surfactant concentration, hence leading to smaller droplet size in the prepared nanoemulsion [24]. Similar findings have been reported by Pengon et al. (2018); these authors found that the droplet size of coconut oil nanoemulsion declined from 33 μm to <200 nm when the concentration of the surfactant was increased from 1% to 10% (*w*/*w*) [25], which is in line with our findings. In agreement with this, Liu et al. (2022) revealed that the droplet size of lemon oil nanoemulsion decreased from 133.71 to 75.66 nm with increasing surfactant concentration [26]; we used a surfactant in our study that enhanced the characteristics of the oil in a similar manner. 

The PDI is a measure of droplet size homogeneity [27,28]. We found that the PDI of the hemp seed oil NEFs ranged from 0.249 ± 0.06 to 0.493 ± 0.02 (Table 4); the calculated range was less than 0.5, indicating a narrow size distribution and a homogenous distribution of droplet size [29]. Our results are in agreement with those reported by Mahdi et al. (2022), who reported that the range of PDI values in cranberry seed oil NEFs was less than 0.5 [30]; a similar range was obtained in the current study.

On the other hand, ZP is the electric potential that exists in the interface that separates the droplets of the internal phase from the external phase on nanoemulsion [31]. As shown in Table 2, the ZP of all hemp seed oil NEFs ranged from −10.32 ± 1.35 mV to −21.94 ± 1.13 mV, see Table 4. The high value of ZP indicates higher physical stability due to a lower tendency of droplet aggregation [7]. The high concentration of LA found in the composition of hemp seed oil may be a direct reason for the high surface negative charges of oil bodies [32]. Additionally, the establishment of a hydrogen bond between the water molecules and oxyethylene group of Tweens may result in the selective adsorption of hydroxyl ion at the oil/water interface, resulting in a negative charge [33]. It was found that ZP was increased by increasing the Tween 80 concentration. These results may be explained by the increase in Tween 80 concentration resulting in smaller droplet size and higher surface area of oil droplets and hence increasing the surface charge. 

### 2.4. Measurement of pH of Hemp Seed Oil Nanoemulsion Formulations

**Figure 2:**
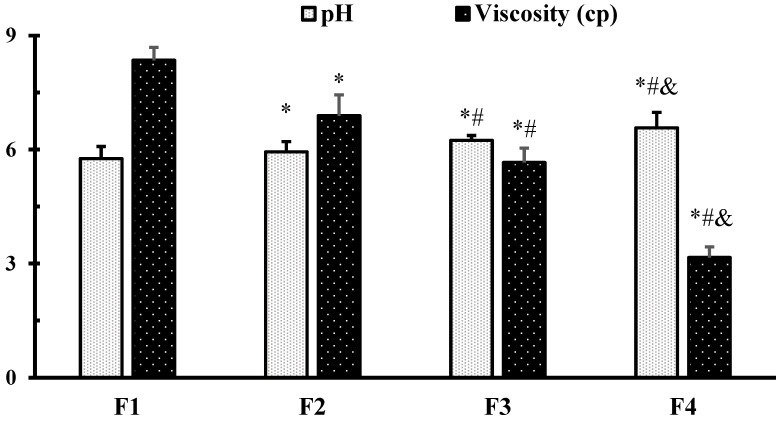
The pH and viscosity of hemp seed oil nanoemulsion formulations. *: versus formulation 1 (F1), #: versus F2, and &: versus F3 at *p* < 0.05.

The pH of hemp seed oil NEFs was measured using a pH meter, and it was found that it ranged from 5.76 ± 0.32 for NEF#1 to 6.57 ± 0.41 for NEF#4 (Figure 2). Similar results were obtained by a Lewinska, who found that the pH value of Cannabidiol oil nanoemulsion was in the range (5.3–6.7) [31]. It was found that the pH of the NEFs increased with increasing concentration of Tween 80, which may be due to the higher stability of the nanoemulsion in the presence of a higher concentration of surfactant.

### 2.5. Viscosity Measurement of Hemp Seed Oil Nanoemulsion Formulations

The viscosity of a nanoemulsion is a very important physical property that affects pourability and drug absorption [34]. As shown in Figure 2, the viscosity of the prepared nanoemulsion formulation ranged from 3.16 ± 0.28 cP for NEF#4 to 8.35 ± 0.34 cP for NEF#1. Lower viscosity was related to smaller droplet size, which may be attributed to the higher surfactant concentration [35]. Similar results were reported by Demisli et al. (2020), who documented that the viscosity of the nanoemulsions was affected by the droplet size [36]. 

### 2.6. Electrical Conductivity Measurement of Hemp Seed Oil Nanoemulsion Formulations

**Figure 3:**
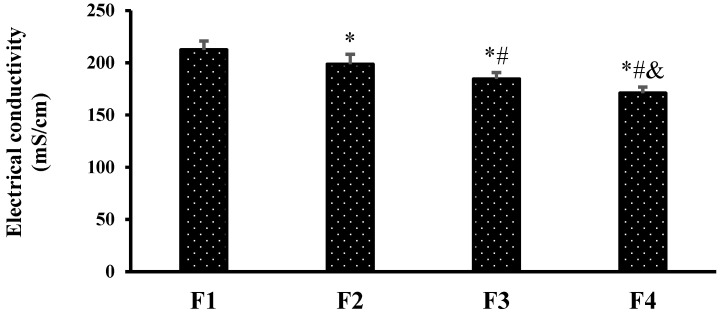
The electrical conductivity of hemp seed nanoemulsion formulations. *: versus formulation 1 (F1), #: versus F2, and &: versus F3 at *p* < 0.05.

The electrical conductivity of the prepared NEFs was in the range of 170.93 ± 5.79 mS/cm for NEF#4 to 212.35 ± 8.46 mS/cm for NEF#1 (Figure 3). It was found, at fixed oil concentration, that the electrical conductivity decreased with increasing surfactant concentration, which may be attributed to the lower water % (*v*/*v*) concentration. Similar results were obtained by Yousef et al. (2019), who found that electrical conductivity was affected by the water content [37].

### 2.7. Selection of Best Hemp Seed Oil Nanoemulsion Formulation

Based on the results of droplet size, ZP, PDI, viscosity, pH, and electrical conductivity, NEF#4 was selected as the optimal formulation to perform the in vivo hepatoprotective activity. 

#### The Surface Morphology of NEF#4

**Figure 4:**
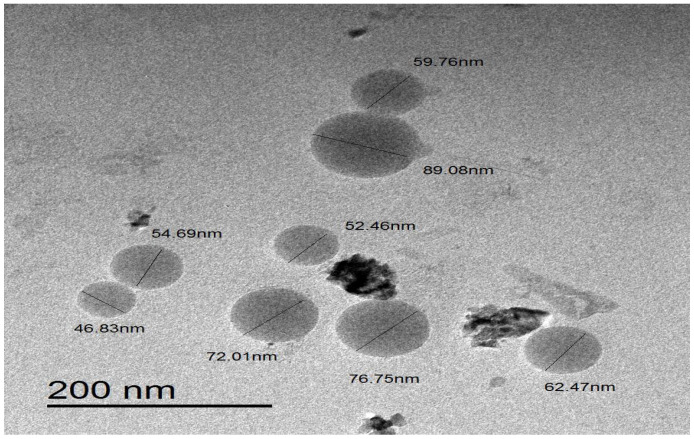
The transmission electron microscopy image of NEF#4.

As shown in Figure 4, the TEM image of the best formulation, NEF#4, presents a spherical appearance and good distribution, with a small droplet size in the nano range, as measured by zetasizer. This result is in good agreement with Liu et al. (2022), who found that the morphology of lemon oil nanoemulsion appeared to be spherical in shape [26]. Additionally, Maccelli et al. (2019) found that the surface morphology of the prepared NEF from *Satureja montana* L. essential oils appeared to be spherical in shape with sizes corresponding to those revealed by DLS analysis [38].

### 2.8. Protective Effect of NEF4 on Fatty Degeneration and Insulin Resistance in Rats

In the current study, feeding male rats with high-fat (HF) diet produced the features of experimental NASH. The NASH control group showed a significant increase in body weight gain (%), as well as significant increments in fasting blood glucose (FBG), serum insulin, and homeostasis model assessment index for insulin resistance (HOMA-IR index) versus rats fed with normal diet (Table 5). The rats that received supplement with hemp seed oil showed amelioration in most of the features of experimental NASH. Moreover, the rats that received supplement of NEF#4 showed better improvements in the aspects of NASH compared to those treated with the ordinary hemp seed oil (Table 5).

Previous studies have shown similar results, confirming that feeding with HF diet results in different aspects of NASH and steatosis. Zaitone et al. (2011) confirmed that feeding rats with HF diet led to significant increments in body weights, HOMA-IR index and serum lipids, in addition to greater liver index, liver enzyme activities and liver steatosis [39]. The authors of this paper confirmed that the two-hit hypothesis (IR and inflammation) successfully explains NASH progression, which is similar to the present situation in the current rat experiment.

Regarding the liver abnormalities, the serum liver enzyme activities (ALT and AST) in the NASH control group were elevated significantly (Table 6). The NASH + hemp seed oil group showed lower liver enzyme activities compared to the NASH control group. Further reductions in liver index and enzyme activities were observed in the NASH+ hemp seed oil NEF#4 (Table 6).

### 2.9. Protective Effect of NEF4 on Liver Histopathology in Rats

**Figure 5:**
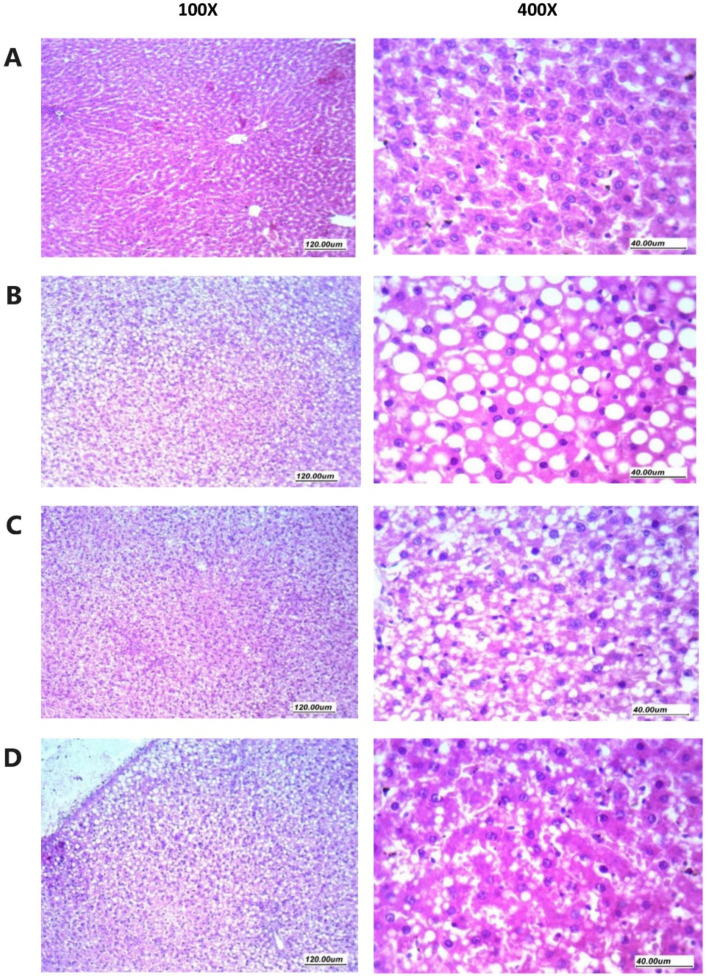
Histopathology of rat liver specimens stained with hematoxylin and eosin. (**A**) Sections from normal liver tissues showing preserved architecture, hepatocytes arranged in thin plates separated by patent sinusoids. Hepatocytes have having homogenous eosinophilic cytoplasm, with regular nuclei. (**B**) Sections from liver tissues in NASH control group showing disturbed architecture, hepatocytes arranged in thick plates with marked steatosis (macro vesicular) presented as large cytoplasmic clear regular vacuole, with obscured sinusoids in-between. Nucleus is pushed to one side of the cells with regular or compressed contours. (**C**) Sections from liver tissues from NASH + hemp seed oil group showing moderately obscured sinusoids and predominantly preserved architecture, hepatocytes showing moderate steatosis (micro vesicular) with small to moderate-sized cytoplasmic clear vacuoles and regular vesicular nuclei. (**D**) Sections from liver tissues from the NASH + NEF#4 of hemp seed oil group showing predominantly preserved architecture, and patent sinusoids, hepatocytes arranged in thin plates, hepatocytes showing mild focal steatosis (micro vesicular) with a small number of scattered small-sized cytoplasmic clear vacuoles and regular vesicular nuclei.

Histologic analysis of H&E-stained liver tissues obtained from normal control (normal diet) mice revealed normal hepatic histology with preserved architecture and intact polygonal hepatocytes having abundant acidophilic cytoplasm and regular nuclei without any significant fat accumulation (Figure 5A). In contrast, sections from the NASH control group showed severe diffuse (>50% of hepatocytes) macrovesicular steatosis in the form of large cytoplasmic fat vacuoles displacing and compressing the nucleus together with narrowed sinusoids and disturbed hepatocyte arrangement (Figure 5B). However, no evidence of lobular or portal inflammation, hepatocyte ballooning, or significant fibrosis was detected. NASH + hemp seed oil group showed relatively preserved hepatocyte architecture and moderate grade microvesicular steatosis mainly in the mid-zone hepatocytes (Figure 5C). Livers from NASH + hemp seed oil NEF#4 group showed approximately normal histologic structure with only mild focal microvesicular steatosis (Figure 5D).

**Figure 6:**
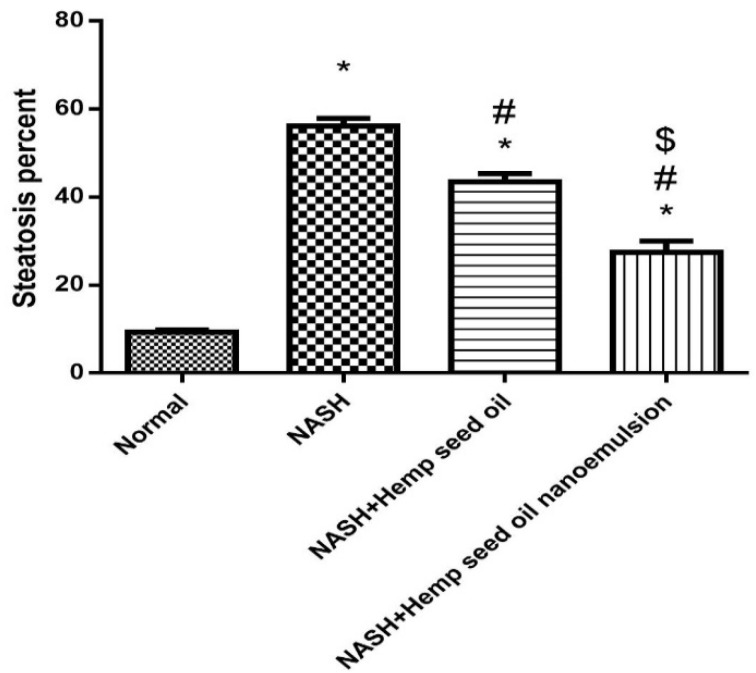
Steatosis score in the hepatic tissues stained with hematoxylin and eosin. Steatosis areas were measured in each microscopic image by labeling the fatty spaces by ImageJ software in images captured at ×400 magnification. Data were processed for statistical analysis using the one-way ANOVA and then Bonferroni’s test for pair-wise comparison at *p* < 0.05. Data are expressed as means ± SD. * Different from normal group, # Different from the NASH group. $ Different from NASH+ hemp seed oil group at *p* < 0.05.

The steatosis score image analysis results indicated that the hepatic fat content in NASH control rats was markedly and significantly higher than in the normal group. The NASH + hemp seed oil group showed significantly lower hepatic fat accumulation; however, the most significant decline in the steatosis degree was detected in livers from NASH + hemp seed oil NEF#4 group (Figure 6).

**Figure 7:**
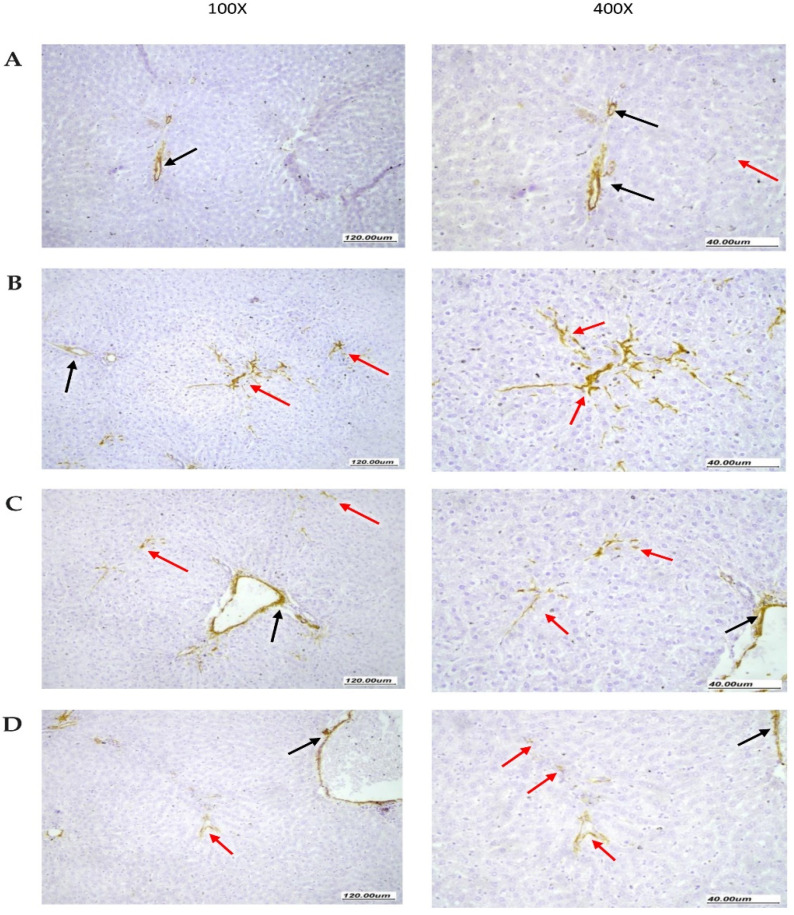
Immunohistochemistry for alpha-smooth muscle actin in rat livers. (**A**) Normal liver tissue showing SMA staining limited to vascular walls (black arrow). The surrounding liver parenchyma shows no SMA staining (red arrow). (**B**) Liver tissues from NASH control group showing moderate SMA staining of stellate myofibroblast cells (red arrow) extending between liver cells within liver parenchyma with arborizing branching pattern between hepatocytes along sinusoidal walls. There are scattered vessels showing staining at periphery (black arrow). (**C**) Liver tissues from the NASH + hemp seed oil group showing focal moderate SMA staining of stellate myofibroblast cells (red arrow) extending early between liver cells within liver parenchyma. There are scattered vessels showing staining at the periphery (black arrow). (**D**) Liver tissues from the NASH + NEF#4 hemp seed oil group showing minimal very focal weak SMA staining of a very small number of myofibroblast cells (red arrow), minimally extending between liver cells within the liver parenchyma. There are scattered vessels showing staining at the periphery (black arrow).

Figure 7 demonstrates the immunohistochemical staining for α-SMA in liver specimens. The normal group showed narrow staining area that was limited to vascular walls while parenchyma was not stained (Figure 7A). However, the NASH control group showed moderate staining in stellate cells with arborizing branching pattern between hepatocytes along sinusoidal walls. Additionally, scattered vessels showed stained myofibroblasts (Figure 7B). In the NASH + hemp seed oil group, liver specimens showed focal moderate SMA staining of stellate myofibroblast cells extended between liver cells within liver parenchyma and scattered vessels showing staining at periphery (Figure 7C). The liver specimens from the NASH + hemp seed oil NEF4 group showed minimal very focal weak SMA staining of very few myofibroblast cells, with minimal extension between liver cells within liver parenchyma, and there were scattered vessels showing staining at the periphery (Figure 7D).

### 2.10. Protective Effect of NEMF4 on Hepatic Free Fatty Acids and Oxidation Parameters

**Figure 8:**
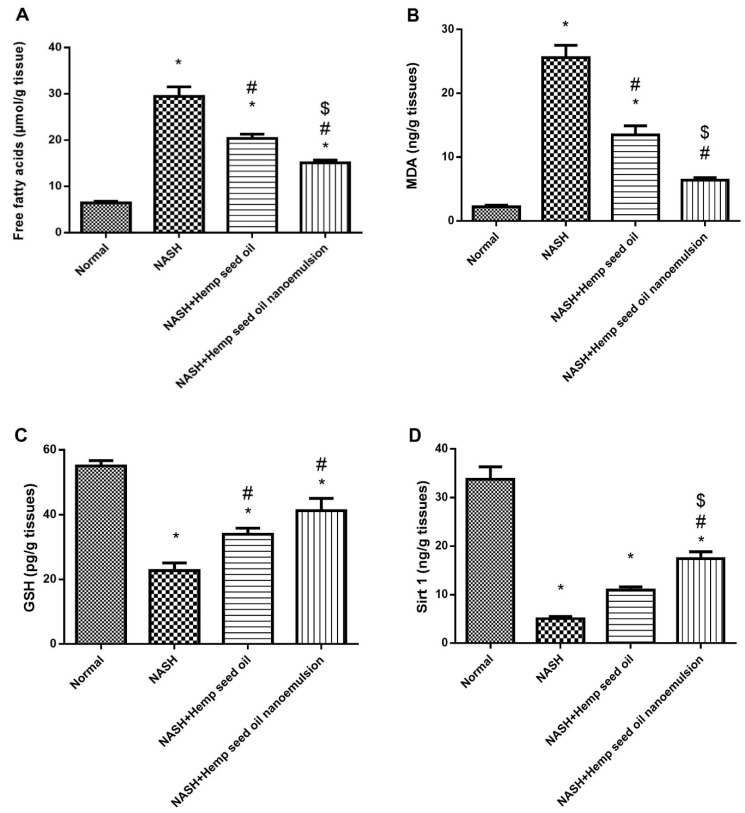
Effect of hemp seed oil and NEF4 on the hepatic level of free fatty acids and oxidation parameters. (**A**) Free fatty acid, (**B**) MDA, (**C**) GSH, and (**D**) Sirt1 levels of the experimental groups. Data were processed for statistical analysis using one-way ANOVA and then Bonferroni’s test for pair-wise comparison at *p* < 0.05. Data are expressed as means ± SD. * Different from normal group, # Different from the NASH group. $ Different from NASH + hemp seed oil group at *p* < 0.05.

Hepatic homogenates were used for measuring FFAs, MDA, GSH and Sirt1. The level of hepatic FFAs and MDA was significantly elevated (4.5-fold and 11.65-fold) in the NSAH group in comparison with the normal group. Treatment with hemp seed oil or its NEF4 produced significant reductions in these two markers (Figure 8A,B). Interestingly, treatment with the hemp seed oil NEF4 produced significant reductions in hepatic FFAs and MDA compared to the NASH control and the NASH + hemp seed oil group (Figure 8A,B).

Regarding the hepatic GSH and Sirt1 values, the NASH control group showed lower levels of these 2 markers compared to the normal group. Treatment with hemp seed oil improved the hepatic level of GSH and Sirt1 compared to NASH control group. Further, treatment with the hemp seed oil NEF#4 produced significant increases in hepatic GSH and Sirt1 compared to the NASH control (Figure 8C,D). The difference in GSH between the NASH + hemp seed oil group and NASH + hemp seed oil NEF#4 was statistically significant.

Previous studies have highlighted elevations in hepatic FFAs and MDA following HF diet feeding in NASH models [40] and human patients [41]. Some data have indicated that dietary fatty acid-induced NASH was normalized after losing trans-FAs from hepatic lipid pools [42], and others have proposed that saturated FAs may be considered an intrinsic second hit affecting the liver, hence hastening NASH development [40]. 

Liu et al. provided evidence that FFAs are linked to the progression of non-alcoholic liver injury in HF-diet-induced obesity; hence, the efforts made to prevent the progression of non-alcoholic liver injury should be wisely forwarded for eliminating the burden of FAs either those transported to the liver or those biosynthesized within the liver [43]. In agreement with our results and the increase in oxidative stress markers, Sumida et al. documented that free radicals and oxidative stress are primary factors in NASH pathology and highlighted the benefit of using antioxidants for mitigation of its progression [44]; this is similar to our results, which indicated greater hepatic MDA and lower GSH.

So far, the working model of NAFLD proposes the “two-hit hypothesis”. IR, which increases food intake and hepatic lipogenesis, may result in accumulation of triglycerides and FFAs in the liver. However, lipid peroxidation, and inflammation may eventually lead to hepatocyte damage and fibrosis.

## 3. Materials and Methods

### 3.1. Chemicals and Drugs

Hemp seed oil was purchased from Nutiva Company (213 West Cutting Blvd Richmond, CA 94804, USA). Pork fat was purchased from the local market. Cholesterol was obtained from GFS chemicals and reagents (Powell, OH, USA), while bile salts were supplied by SAS Chemicals Co. (Mumbai, India).

### 3.2. Characterization of the Content of Hemp Seed Oil

The working sample of hemp seed oil was characterized with respect to its composition of saponifiable and unsaponifiable contents according to a method previously described by El Sayed et al. (2020) with slight modifications [45]. Briefly, about 2 g of hemp seed oil was refluxed with 15 mL ethanolic solution of 10% potassium hydroxide solution in a refluxing spherical flask connected to a water-cooled condenser heated in a water bath at a temperature of 90–100 °C for 5 h. The refluxed mixture was then cooled to room temperature, mixed with 20 mL of distilled water, and transferred to a separating funnel. The resulting aqueous mixture was extracted three times each with 20 mL of petroleum ether. Sodium chloride (1–2 g) was added to the first extraction time to facilitate phase separation. The organic extracts were pooled for preparation of unsaponifiable matter sample while the aqueous phase was retained for preparation of saponifiable matter samples.

For the unsaponifiable matter sample, the pooled organic layer extract was washed five times with 100 mL distilled water. Then, the organic layer was filtered over anhydrous calcium chloride sample and the filtrate was evaporated at 40 °C under reduced pressure (−600 to −800 mBar) until the volume of residue was less than 2 mL. The residue was then transferred into an Eppendorf tube and evaporation was continued until complete evaporation of the solvent had been achieved. The residue was dissolved in hexane, GC grade, and chromatographed by GC-MS to determine unsaponifiable matter composition.

The saponifiable matter portion was methyl-esterified as follows: the aqueous phase from the saponification reaction was acidified by 15 mL of 10% hydrochloric acid solution in a separating funnel and the free fatty acids extracted using three portions of petroleum ether, each of 20 mL. The petroleum ether extract portions were pooled and filtered over anhydrous calcium chloride and then evaporated at 40 °C under reduced pressure (−600 to −800 mBar) to remove petroleum ether. The residue was dissolved in 50 mL of methanol and 5 mL of concentrated sulfuric acid and the mixture was refluxed for 2 h at 90–100 °C for esterification. The mixture was then extracted with three portions of n-hexane, each of 20 mL. The hexane extracts were pooled and evaporated at 40 °C under reduced pressure (−600 to −800 mBar) until the residue volume was less than 2 mL. The residue was then transferred into an Eppendorf tube and evaporation was continued until complete evaporation of the solvent had been achieved. The residue was dissolved in hexane, GC grade and chromatographed by GC-MS for determination of fatty acid methyl ester composition in saponifiable matter of the oil. 

The contents of both the unsaponifiable and saponifiable samples were chromatographed with mass spectra acquisition using Shimadzu GCMS-QP2010 SE (Shimadzu Inc., Koyoto, Japan), applying split injection ratio of 1:30 and injector temperature of 280 °C. The chromatographic separation of compounds was performed using Rtx-5MS fused bonded capillary (30 m length × 0.25 mm i.d. × 0.25 μm film thickness) (Restek, Bellefonte, PA, USA). The column oven temperature program started with an isothermal segment of 3 min at 50 °C for 3 min and was then increased to 300 °C with a rate of 5 °C/min, then held isothermally at 300 °C for 10 min. Injector temperature was 280 °C. The carrier gas was pure helium at a flow rate of 1.37 mL/min. The mass detector settings were configured as follows: filament emission current at 60 mA; ionization voltage at 70 eV; ion source temperature 220 °C.

Analyzed compounds were identified by comparison of MS fragmentation spectra with standard NIST library. Composition of saponifiable and unsaponifiable parts was determined using the Area % calculation method of the resolved compounds. 

### 3.3. Preparation of Hemp Seed Oil Nanoemulsion

**Figure 9:**
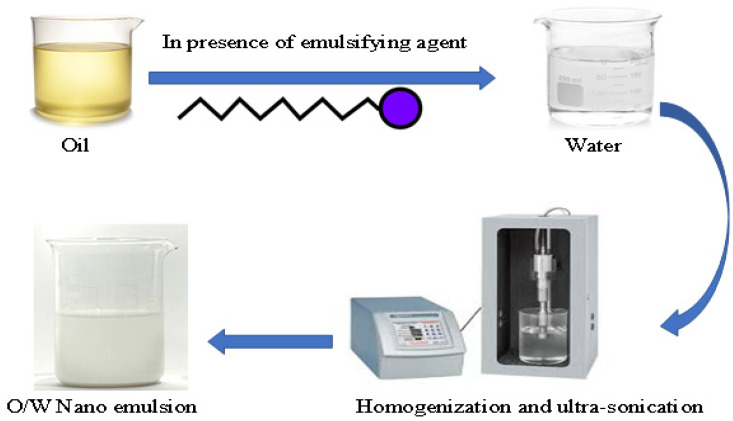
A diagram illustrating preparation of the oil/water nanoemulsion preparations.

Four hemp seed oil (O/W) nanoemulsion preparations were formulated using the high-pressure homogenization technique (Figure 9). We dissolved the accurate volume of surfactant and co-surfactant in the accurate volume of water in a small beaker [46]. An accurate volume of oil was added to the aqueous phase drop while homogenizing using a high shear homogenizer at 20,000 rpm until the total volume of oil had been homogenized, and then homogenization was continued for another 10 min [47]. The prepared NEFs were stored at 5 °C for 24 h before further investigation [48,49]. 

#### 3.3.1. Measurement of the Droplet Size, Zeta Potential and Polydispersity Index of the O/W Nanoemulsion Preparations of Hemp Seed Oil

The droplet size, ZP, and PDI, of all the prepared hemp seed oil o/w NEFs were determined using a Malvern Zetasizer (Malvern Instruments Ltd., Malvern, UK), applying the dynamic light scattering technique [27]. To measure the droplet size and ZP, we diluted a sample of each formulation (1:100) with distilled water at 25 °C [50,51]. ZP values of the nanoemulsions were estimated from the electrophoretic mobility of oil droplets [52]. All measurements were performed in triplicate. 

#### 3.3.2. Evaluation of the pH of the Hemp Seed Oil Nanoemulsions

The pH value of the prepared nanoemulsion formulations (NEFs) was measured by a pH meter (Jenway, Staffordshire, UK) after homogenizing with water (1:9) [53,54]. The measurements were performed in triplicate at room temperature. 

#### 3.3.3. Viscosity Evaluation

The viscosity is one of the important physical properties of nanoemulsions [55]. The viscosity of the nanoemulsion formulations was determined using Brookfield rheometer and a Brookfield viscometer R/S+RHEOMETER (Brorokfield Inc., Brookfield, MA, USA) with spindle number CC 14 rotating at 5 rpm at a fixed temperature = 37 °C for a run time equal to 10 s [56]. 

#### 3.3.4. Measuring Electrical Conductivity of the NEFs

The electrical conductivity of the NEFs was measured utilizing a digital conductometer (HANNA Instrument, H1255, Cluj-Napoca, Romania) at room temperature. A conductometer was employed to determine the conductance of nanoemulsion [57,58]. The test was performed in triplicate.

#### 3.3.5. The Selection of the Best Nanoemulsion Formulation

The best NEF was chosen to be utilized in the in vivo rat study; this selection was based on the smallest droplet size and the highest physical stability.

##### Transmission Electron Microscopy of the Best Formulation (TEM)

The surface morphology of the best formulation of the prepared nanoemulsion (NEF4) was studied using a jeol transmission electron microscope (JTEM model 1010, JEOL^®®^, Tokyo, Japan). One drop of NEF4 was added to a copper-coated collodion grid after appropriate dilution and allowed to dry. The sample was stained by addition of uranyl acetate and pictured by TEM after drying at room temperature [26].

### 3.4. In Vivo Study of Hepatoprotective Activity

#### 3.4.1. Experimental Animals

Adult male Wistar rats were purchased from the Egyptian Organization for Biological Products and Vaccines. Rats’ body weight ranged from 120 to 150 g at the beginning of the study. Feeding material and water were allowed ad libitum. Strict care, hygiene and normal dark/light cycle were maintained for a healthy environment. The study protocol followed the ethical guidelines of the Research Ethics Committee at the Faculty of Pharmacy, Suez Canal University (202112RA3).

#### 3.4.2. Experimental Design and Grouping

Rats were randomly allocated to 4 groups, 5 rats in each group, as follows:

Group I (Normal rats): Rats were maintained on the standard rat chow diet.

Group II (NASH rats): Rats were maintained on a HF diet for 16 weeks. The diet composition was as follows: 87.7% basal rat chow mixed with 10% pork fat, 2% cholesterol, and 0.3% bile salts. The HF diet was prepared every 5 days and divided into parts which were kept at 4 °C and left at room temperature for 1 h before use [59]. 

Group III (NASH + hemp seed oil): Rats were fed with the HF diet and received hemp seed oil by oral gavage at 2 mL per rat, three times per week [60] from week 9 to end of week 16.

Group IV (NASH + hemp seed oil in nanoemulsion): Rats were fed with the HF diet and received hemp seed oil NEF#4 (as 2 mL/rat from week 9 to end of week 16) by oral gavage.

#### 3.4.3. Tissue Collection and Sample Preparation

After finishing the study protocol at the end of week 16, the rats’ body weights were measured in order to calculate the body weight gain % throughout the study period. After overnight fasting, rats were anesthetized and sacrificed by applying the cervical dislocation procedure. We withdrew blood samples from the retro-orbital plexus and kept them aside for 30 min and then centrifuged the samples at 1500× *g* for 10 min. Serum samples were isolated and kept at −20 °C until use in different assays. Moreover, the livers were dissected and washed out of blood, dried on a filter paper and weighed [61]. 

#### 3.4.4. Measuring the Fasting Blood Glucose and Insulin and Calculation of HOMA-IR Index

A spectrophotometric kit from Spinreact (Girona, Spain) was used for measuring fasting blood glucose while insulin was determined using an enzyme-linked immunosorbent assay (ELISA) Rat Insulin Kit (Cat. No. RK03773, Woburn, MA, USA) based on competitive inhibition enzyme immunoassay. The minimum sensitivity of the assay was 50.2 pg/mL, according to the determination protocol of the manufacturer. Utilizing an ELISA reader (Europe S.A., Woluwe-Saint-Pierre, Belgium), the optical density of the final reaction product was measured at 450 nm. IR was determined using the HOMA-IR index. We utilized the formula published by Matthews et al. “HOMA-IR index = [fasting glucose (mmol/L) × fasting insulin (μU/mL)]/22.5)” [62]. 

#### 3.4.5. Determination of Serum Liver Enzyme Activities

For evaluation of liver function, serum aspartate aminotransferase (AST), alanine aminotransferase (ALT), and alkaline phosphatase (ALP) activities were determined by enzymatic colorimetric assay kits (Spinreact, Girona, Spain) using a spectrophotometer (UV-1601-PC; Shimadzu, Japan) according to the manufacturer’s instructions for each kit.

#### 3.4.6. Determination of Sirt1 by Enzyme-Linked Immunoassay Kits

One hundred milligrams of frozen liver samples were employed to measure Sirt1. Tissues were rinsed with phosphate-buffered saline (PBS), homogenized in 1 mL of PBS and stored at −20 °C. We performed two freeze-thaw cycles for breaking the cell membranes and then put the homogenates in a centrifuge at 2000× *g* for 8 min. The supernatants were then removed. Supernatant aliquots were stored at −20 °C, then SIRT1 concentration was estimated using a rat Sirt1 quantitative sandwich ELISA kit (Cat. No. MBS060720, MyBioSource Inc., San Diego, CA, USA) following the producer’s instructions. Sensitivity of the applied ELISA kit was 0.1 ng/mL within a detection range equals 0.625–20 ng/mL. The optical density of the reaction was measured using an ELISA reader at 450 nm.

#### 3.4.7. Assay of Free Fatty Acids, Malondialdehyde and GSH

Free fatty acids were determined using a rat free fatty acid (FFA) ELISA kit (Cat. No. MBS014345, MyBioSource Inc., San Diego, CA, USA) based on quantitative sandwich immunoassay and optical density measurement at 450 nm by ELISA reader. The kit sensitivity was 5.0 μmol/L within a detection range of 31.2–1000 μmol/L following the manufacturer’s instructions. MDA was determined by a rat malondialdehyde (MDA) competitive ELISA kit (Cat. No. LS-F28018, Lifespan Biosciences, Seattle, WA, USA) according to the manufacturer’s instructions. GSH determination was performed utilizing a rat reduced glutathione ELISA kit (Cat. No. E02G0367, Shanghai BlueGene Biotech, Shanghai, China), applying the quantitative sandwich immunoassay technique according to the manufacturer’s protocol. All ELISA measurements of optical density were performed using an ELISA reader (Europe S.A., Woluwe-Saint-Pierre, Belgium) at 450 nm.

#### 3.4.8. Histopathological Examination of Liver Tissue

The upper parts of the right lobes of the livers were taken, washed in saline, and dried by filter paper in fragments with a thickness of about 1 mm. Fragments were fixed in 200 mL of 10% neutral buffered formalin for 24 h, washed, dehydrated, immersed in xylene, and then embedded in paraffin blocks and processed into 5 μm sections for light microscopic examinations by hematoxylin and eosin stain (H&E) [63]. Sections were examined using a light microscope. Images were captured using a calibrated standard digital microscope camera (Tucsen^®®^ ISH100, Fuzhou, China) using an Olympus^®®^ CX23 (Tokyo, Japan) microscope with a resolution of 10 MP (megapixels) 3656 × 2740 pixel. All H&E-stained slides were captured at an original magnification of 100× and 400× (Objectives 10× and 40× respectively) using a UIS optical system (Universal Infinity System, Olympus^®®^, Tokyo, Japan). Steatosis areas were measured in each microscopic image by labeling the fatty spaces using ImageJ software in images captured at ×100 magnification [64]. Briefly, each image was dragged and dropped into the ImageJ program and adjusted as an 8-bit image. The threshold was adjusted in the RGB image style. This step makes the steatosis appear as circles filled with black. From the “Process” menu, we selected “analyse particles”, then the surface area of all rounded regular shapes filled with black (i.e., steatosis) was measured in pixels, and the results were then displayed in the results table, as percent (%) in relation to the surface area of the whole image field (which measures 979 × 734 um). 

#### 3.4.9. Immunohistochemistry and Image Analysis

Paraffin sections of hepatic tissues were sectioned at 4 µm, de-waxed with xylene, and irrigated for 30 min with 3% hydrogen peroxide in methanol in the dark to inactivate endogenous peroxidase. Tissue sections were dehydrated through graded alcohols then put on slides coated with poly-L-lysine. For routine antigen retrieval, tissue sections were then immersed in citrate buffer (0.01 M, pH 6.0) and put in a microwave for 15 min. After that the sections were incubated with α-smooth muscle actin (α-SMA) rabbit polyclonal antibodies (A7248, 1:100, ABclonal, Woburn, MA, USA) at 4 °C for 24 h. Chromogen DAB (3, 3′-diaminobenzidine tetrahydrochloride) from Genemed Biotechnologies (San Francisco, CA, USA) was added to visualize the reaction. After rinsing off DAB, Mayer’s hematoxylin was used for counterstaining. Slides were examined by a light microscope and photographs were captured and analyzed (the percent of DAB-colored area) using ImageJ software developed by the National Institute of Health (Bethesda, MD, USA) [65].

### 3.5. Data Collection and Statistical Analysis

Statistical analysis was performed using GraphPad Prism (version 6.00, GraphPad Software Inc., San Diego, CA, USA). Data were collected and tabulated in Microsoft Excel and checked for Gaussian distribution using the K-S test and then expressed as means and standard deviations of the mean (SD). All data were two-tailed and analyzed by applying the one-way analysis of variance (ANOVA) test and then a pair-wise comparison was performed to explore the differences between every pair of groups using Bonferroni’s test. The differences were considered significant if the *p* value was <0.05. 

## 4. Conclusions

The current study highlighted the usefulness of hemp seed oil in alleviating NASH symptoms in rats. Further, the hemp seed oil NEF#4 produced superior biological activity—as indicated by the biological indicators (62% decline in HMOA-IR index, liver enzymes and ~50% decline in hepatic FFAs) and histopathology (~50% decrease in steatosis % and a decline in fibrosis)—against experimental NASH when compared to the original formula of the hemp seed oil. Hence, the current study highlighted that a nanoemulsion formulation of hemp seed oil is useful in enhancing its biological activity and is promising for more experimental trials in animals.

## Figures and Tables

**Table 1:** Fatty acid methyl esters determined in saponifiable matter of the working hemp seed oil sample.

Fatty Acid Methyl Esters	% *w*/*w* *	Base Ion (*m*/*z*)
(9Z,11E,13E)-octadeca-9,11,13-trienoic acid methyl ester	0.05	79.05
(Z)-Methyl heptadec-9-enoate	0.07	55.05
10-Heptadecen-8-ynoic acid, methyl ester, (E)	0.15	79
13-Docosenoic acid, methyl ester, (Z)	0.06	55.05
7,10,13-Eicosatrienoic acid, methyl ester	0.17	67.05
9,11-Octadecadienoic acid, methyl ester, (E,E)	0.75	95.05
9,12,15-Octadecatrienoic acid, methyl ester, (Z,Z,Z)	73.7	79.05
9-Hexadecenoic acid, methyl ester, (Z)	0.23	55.05
*cis*-11-Eicosenoic acid, methyl ester	0.98	55.05
Docosanoic acid, methyl ester	0.61	74
Heptadecanoic acid, methyl ester	0.08	74
Hexadecanoic acid, methyl ester	7.66	74.05
Methyl γ-linolenate	3.98	79.05
Methyl 18-methylnonadecanoate	1.45	74.05
Methyl stearate	3.68	74
Tetracosanoic acid, methyl ester	0.3	74
Tricosanoic acid, methyl ester	0.11	74

* % *w*/*w* from the saponifiable matter of working hemp seed oil sample.

**Table 2:** Compounds determined in unsaponifiable matter of the working hemp seed oil sample.

Compound	% *w*/*w* *	Base Ion (*m*/*z*)
Benzene, (1,3,3-trimethylnonyl)–	1.84	105.1
Benzene, (1-butylheptyl)–	1.18	91.05
Benzene, (1-butyloctyl)–	1.15	91.1
Benzene, (1-ethylnonyl)–	1.01	91.05
Benzene, (1-methyldecyl)–	1.56	105.1
Benzene, (1-pentylheptyl)–	1.22	91.1
Benzene, (1-pentyloctyl)–	1.04	91.1
Benzene, (1-propyloctyl)–	1.23	91.05
Decyl oleate	1.16	57.05
Diisooctyl phthalate	3.98	149.1
Dodecane	3.83	57.05
Eicosane	3.49	57.05
Glycidyl (Z)-9-Heptadecenoate	13.72	55.05
Glycidyl palmitate	2.02	57.05
Heneicosane	1.07	57.1
Heneicosane	1.16	57.05
Heneicosane	1.43	57.1
Heneicosane, 10-methyl–	0.93	57.1
Heptadecane	1.08	57.1
Hexatriacontane	2.43	57.05
Hexatriacontane	1.32	57.05
Hexatriacontane	1.73	57.1
Oleoyl chloride	14.28	55.05
Oxalic acid, cyclohexylmethyl octadecyl ester	1.13	97.1
Silane, diethylheptyloxyoctadecyloxy–	4.37	441.4
γ-Sitosterol	4.95	43.05

* % *w*/*w* from the unsaponifiable matter of working hemp seed oil sample.

**Table 3:** Composition of hemp seed oil nanoemulsion formulations.

Formulation No	Hemp Seed Oil % (*v*/*v*)	Surfactant (Tween 80% *v*/*v*)	Propylene Glycol % (*v*/*v*)	Water % (*v*/*v*)
NEF#1	40	2.5	5	52.5
NEF#2	40	5	5	50
NEF#3	40	7.5	5	47.5
NEF#4	40	10	5	45

NEF#1–NEF#4: nanoemulsion formulas 1–4.

**Table 4:** Characterization of hemp seed oil nanoemulsion formulations.

Formulations No	Droplet Size (nm)	PDI	Zeta Potential (mV)
NEF#1	140.9 ± 5.62	0.493 ± 0.02	−10.32 ± 1.35
NEF#2	121.3 ± 3.41	0.438 ± 0.01	−12.67 ± 2.68
NEF#3	98.6 ± 9.54	0.312 ± 0.03	−19.26 ± 2.92
NEF#4	80.7 ± 3.96	0.249 ± 0.06	−21.94 ± 1.13

NEF#1–NEF#4: nanoemulsion formula 1–4.

**Table 5:** Effect of the hemp seed oil NEF#4 on body weight, blood glucose, insulin, and insulin resistance index in rats with NASH.

	Δ BWt. %	Glucose (mg/dL)	Insulin (µIU/L)	HOMA-IR Index
Normal	18.59 ± 6.76	76.4 ± 2.30	12 ± 2.92	1.99 ± 0.38
NASH control	51.43 ± 8.17 *	96.2 ± 2.77 *	47.6 ± 6.80 *	11.28 ± 1.42 *
NASH + Hemp seed oil	44.48 ± 3.78 ^#^	84 ± 5.24 ^#^	30 ± 6.04 ^#^	6.35 ± 1.39 ^#^
NASH+ Hemp seed oil NEF#4	32.34 ± 6.74 ^#$^	78 ± 2.23 ^#^	22.2 ± 5.2 ^#$^	4.29 ± 1.11 ^#$^

Δ BWt. % = (Final body weight − baseline body weight/baseline body weight) * 100; HOMA-IR index: homeostasis model assessment–insulin resistance index. Data are expressed as means ± SD. * Different from normal group, ^#^ Different from the NASH group. ^$^ Different from NASH + hemp seed oil group at *p* < 0.05.

**Table 6:** Liver enzyme activities in rats with NASH treated with hemp seed oil and NEF#4.

	Alanine Aminotransferase (IU/L)	Aspartate Aminotransferase (IU/L)
Normal	40.8 ± 3.35	61.6 ± 2.97
NASH control	85.4 ± 7.89 *	97 ± 6.78 *
NASH + Hemp seed oil	62.6 ± 9.76 ^#^	83.6 ± 6.73 ^#^
NASH+ Hemp seed oil NEF4	56 ± 5.48 ^#$^	68.4 ± 5.13 ^#$^

Data are expressed as means ± SD. * Different from normal group, ^#^ Different from the NASH group. ^$^ Different from NASH+ hemp seed oil group at *p* < 0.05.

## Data Availability

Data are available from the corresponding author upon request.
